# Epidemiological and Clinical Changes in RSV-Associated Pneumonia in Children in Mexico Before and During the COVID 19 Pandemic

**DOI:** 10.3390/idr17060139

**Published:** 2025-11-08

**Authors:** Ilen Adriana Diaz-Torres, Isamu Daniel Cabrera-Takane, Fanny Yasmin Ortega-Vargas, Aldo Agustin Herrera-González, Miguel Leonardo Garcia-León, Patricia Bautista-Carbajal, Daniel E. Noyola, Maria Susana Juárez-Tobías, Pedro Antonio Martínez-Arce, María del Carmen Espinosa-Sotero, Verónica Tabla-Orozco, Gerardo Martínez-Aguilar, Fabian Rojas-Larios, Rosa María Wong-Chew

**Affiliations:** 1 Infectious Diseases Research Laboratory, Research Division, Facultad de Medicina, Universidad Nacional Autónoma de México, Mexico City 04510, Mexico; ilentztrs@gmail.com (I.A.D.-T.); isamu.takane7@gmail.com (I.D.C.-T.); fannyortegavargas@gmail.com (F.Y.O.-V.); herrera.aldo24@gmail.com (A.A.H.-G.); mlgl7@unam.mx (M.L.G.-L.); pbautistac@comunidad.unam.mx (P.B.-C.); 2 Centro de Investigación en Ciencias de la Salud y Biomedicina, Universidad Autónoma de San Luis Potosí, San Luis Potosí 78210, Mexico; dnoyola@uaslp.mx; 3 Hospital Central “Dr. Ignacio Morones Prieto”, San Luis Potosí 78290, Mexico; susana.juarez@uaslp.mx; 4 Antiguo Hospital Civil de Guadalajara Fray Antonio Alcalde, Guadalajara 44200, Mexico; pama130@yahoo.com.mx; 5 Hospital General de México “Dr. Eduardo Liceaga”, Mexico City 06720, Mexico; carmenespinosa6@hotmail.com; 6 Hospital Pediátrico de Coyoacán, Mexico City 04000, Mexico; vtablaorozco@gmail.com; 7 Hospital Municipal del niño de Durango, Durango 34105, Mexico; uimec@yahoo.es; 8 Facultad de Medicina y Nutrición, Universidad Juárez del Estado de Durango, Durango 34113, Mexico; 9 Hospital Regional Universitario IMSS Bienestar Colima, Colima 28019, Mexico; frojas@ucol.mx

**Keywords:** children, RSV, Mexico, pneumonia, pandemic, virus, respiratory, incidence

## Abstract

**Background/Objectives:** Respiratory syncytial virus (RSV) significantly affects young children. In 2020, at the beginning of the COVID-19 pandemic, widespread public health measures temporarily interrupted RSV transmission. However, by mid-2021, an atypical resurgence of RSV was observed. The objective of this study was to compare the clinical and epidemiological characteristics of RSV infections in children before and during the second half of the SARS-CoV-2 pandemic in Mexico. **Methods**: A comparative ambispective longitudinal epidemiological study was conducted using two distinct cohorts: one from 2010 to 2013 and another from 2021 to 2023. The study included children under five years of age diagnosed with RSV-related pneumonia. Statistical analyses included Student’s *t*-tests, chi-square tests, and logistic regression to identify risk factors associated with severe pneumonia. Incidence density was calculated as the number of RSV-positive pneumonia cases per 10 new pneumonia admissions per month. **Results**: The mean age of affected children increased from 10 to 15 months. RSV activity began earlier in 2021, emerging during the summer months, and showed a higher incidence than in previous seasons. RSV type B was significantly more common during the pandemic period (58.5% vs. 3.8%), and the proportion of co-infections also increased (60% vs. 39%), indicating a change in the viral landscape. **Conclusions**: These findings indicate a shift in RSV seasonality toward summer and autumn, increased case incidence, and infections in older children. These observations underscore the need for ongoing surveillance to better understand evolving RSV patterns, especially in the context of complex public health scenarios like the COVID-19 pandemic.

## 1. Introduction

Respiratory syncytial virus (RSV) is one of the most common respiratory pathogens affecting young children, but it has also recently been identified as a significant pathogen in the elderly and immunocompromised patients [1]. Manifestations range from mild upper respiratory tract infections to severe lower respiratory tract diseases, such as bronchiolitis and pneumonia, with potential long-term effects including the development of childhood asthma [2]. Risk factors for severe disease include prematurity, congenital heart disease, chronic lung disease or immunodeficiency. Notably, however, a significant number of hospitalizations occur among full-term infants without comorbidities. It is estimated that RSV was associated with 3.6 million hospital admissions and 101,400 deaths in children under 5 years of age globally in 2019 [3]. Morbidity is particularly high among infants younger than six months, who account for approximately 39% of RSV-related lower respiratory tract infection (LRTI) hospitalizations. More than half of all RSV-associated in-hospital deaths occur in children under five years of age, with about 97% of these pediatric deaths taking place in low- and middle-income countries. The hospitalization rate for RSV is 10 times higher than that for influenza. The disease burden is exacerbated by the lack of pharmacological treatments, with supportive care being the standard approach [4]. Currently, available preventive strategies include palivizumab and nirsevimab, as well as maternal vaccination with RSVpreF; however, these measures were not yet available in Mexico during the 2021–2023 study period.

In Mexico, RSV is also one of the main pathogens in LRTI in children; in a study with 1404 children younger than 5 years old with community-acquired pneumonia from 11 hospitals across the country, RSV was identified as the most frequently detected virus, present in 38.1% of cases. It appeared as a single pathogen in 23.7% of cases and in conjunction with other pathogens in 14.4% of cases, with a high prevalence in autumn and winter [5].

At the onset of the COVID-19 pandemic, public health and social measures (PHSMs), including mask use, hand hygiene, social distancing, travel restrictions, school closures and enhanced surveillance measures, were implemented globally. These interventions significantly curtailed the circulation of many viruses, including RSV [1], as seen in studies conducted in England, Japan and Australia, which reported reductions in RSV cases of 99%, 93% and 100%, respectively, during the 2020–2021 winter season [6].

However, by mid-2021, the circulation of other respiratory viruses had resumed. The re-emergence of RSV was atypical, with cases occurring during unusual seasons (summer–autumn) and following patterns that differed from previous years, affecting countries such as the United States, Australia, and Japan [7]. Early RSV epidemics in 2021 saw detection rates surpassing pre-pandemic peaks. In England, for example, the total number of RSV-related respiratory diseases, acute bronchiolitis, and hospital admissions increased by more than 100%, 84% and 10%, respectively. Additionally, shifts in the subtype of RSV prevalence were observed, with a dominance of RSV type B in Australia, contrasting the prior predominance of RSV-A [6].

Given that RSV is primarily transmitted through close contact, public health and social measures (PHSMs) likely had a more pronounced effect on RSV transmission in the pediatric populations, subsequently reducing transmission to other high-risk groups, such as the elderly. In this study, we analyzed the clinical and epidemiological differences in RSV in children during the latter half of the COVID-19 pandemic in Mexico, comparing these findings to data from a prior period (2010–2013) involving children with pneumonia from the same hospitals in Mexico [5].

## 2. Materials and Methods

### 2.1. Study Design

A comparative ambispective longitudinal epidemiological study of two cohorts was conducted to assess RSV incidence, demographic, clinical characteristics, and risk factors for severe pneumonia, both before and during the COVID-19 pandemic.

### 2.2. Data Source

The analysis was based on data from two cross-sectional studies. The first cohort was collected from March 2010 to August 2013, and the second cohort from July 2021 to March 2023. All patients had been hospitalized with a clinical and/or radiological diagnosis of pneumonia (ICD J18.9) and were aged five years or younger. Data were collected using a specially designed format created for the study. The information was obtained from parent or guardian interviews and medical records. It included demographic characteristics, clinical characteristics and risk factors for pneumonia.

Information on in-hospital antibiotic use was available for the 2021–2023 cohort. We operationally defined antibiotic use as the administration of any systemic antibacterial agent during hospitalization; prescriptions consisting exclusively of antivirals (e.g., oseltamivir) were not counted as antibiotic use.

The first study was conducted in 11 hospitals across Mexico, including Nuevo Hospital Civil de Guadalajara, Hospital Regional Universitario de los Servicios de Salud del Estado de Colima, Hospital Pediátrico de Coyoacán, Hospital General de Durango, Hospital General de Mexicali, Hospital Central “Dr. Ignacio Morones Prieto” San Luis Potosí, Hospital para el Niño de Toluca, Hospital General de México “Dr. Eduardo Liceaga”, Hospital de Pediatría del CMNO, Instituto Mexicano del Seguro Social (IMSS), Guadalajara, Hospital de la Niñez Oaxaqueña, and Hospital Infantil de Tlaxcala) [5]. Of the first cohort, 531 cases were included in the analysis due to the availability of complete demographic data, which were used for comparison with the second cohort, assembled from July 2021 to April 2023 in six hospitals (Hospital Central “Dr. Ignacio Morones Prieto” San Luis Potosí, Nuevo Hospital Civil de Guadalajara, Hospital Pediátrico de Coyoacán, Hospital General de México “Dr. Eduardo Liceaga”, Hospital Municipal del Niño de Durango, and Hospital Regional Universitario de los Servicios de salud del Estado de Colima). The second cohort included 248 confirmed RSV-positive cases.

Before 2010, multiplex PCR techniques for identifying respiratory pathogens were not available. In 2010, our research group conducted a study using this technique in children with pneumonia, with recruitment ending in 2013. The comparison of the pandemic period with the 2010 to 2013 period was feasible because six of the same hospitals participated in both studies, and data for comparison was available. According to the Mexican Ministry of Health, the epidemiological behavior of respiratory viruses remained consistent between 2010 and 2019, further justifying the use of the 2010–2013 data as a baseline for comparison [8].

The 2010–2013 dataset used in this study derives from the previously published multicenter study by Wong-Chew et al. [5], which obtained ethics approval at all participating institutions. In that study, parents or guardians provided written informed consent authorizing that the de-identified data could be used for future analyses within a 20-year period after collection. For the present work, a secondary analysis of these de-identified data was conducted in accordance with the approved protocols.

### 2.3. Ethical Statement

The study was approved by the ethics and research committees of all participating institutions (Facultad de Medicina UNAM FM/DI/105/2020 (approved on 11 May 2020), Hospital Civil de Guadalajara 052/20 (approved on 9 July 2020), Hospital Municipal del Niño de Durango 001/2021 (approved on 13 July 2021), Hospital Central Dr Ignacio Morones Prieto 37-21 (approved on 26 May 2021), Hospital General de México Dr. Eduardo Liceaga DI/22/505/05/42 (approved on 29 September 2022), Hospital Pediatrico de Coyoacan 101-011-025-21 (approved on 12 October 2021), Hospital universitario de los servicios de salud del estado de Colima CI 2021/02/CR/PED/130 (approved on 12 November 2021)). Parents or guardians of children diagnosed with pneumonia were invited to have their children participate in the study. Written informed consent was obtained before any procedure was performed.

### 2.4. Case Definition, Socio-Demographic and Hospitalization Data

The case definition for pneumonia included the presence of respiratory symptoms such as respiratory distress, tachypnea, cyanosis, or cough, with or without fever lasting less than one week, and/or an X-ray showing pulmonary infiltrates in children younger than 5 years old. Pneumonia was classified as severe and non-severe according to the WHO definition [9]. Radiographic patterns were categorized as interstitial, micronodular, macronodular, multiple foci, lobar, pleural effusion and mixed by attending pediatricians or site radiologists using predefined diagnostic criteria, and the findings were recorded in the medical charts prior to data entry.

Demographic data, including age, sex, weight, date of diagnosis, date of birth, clinical signs and symptoms, radiographic patterns, hospital area of attendance and risk factors including influenza vaccination, incomplete vaccination schedule (Defined as the absence of one or more doses of the age-appropriate vaccines according to the Mexican National Immunization Schedule), breastfeeding, use of biomass, immunocompromise, co-morbidities and information on household tobacco smoke exposure was collected through structured interviews with parents or guardians.

### 2.5. Anthropometric Assessment

Anthropometric indicators were analyzed using the 2006 WHO Child Growth Standards through the WHO Anthro software (Version 2006, WHO, Geneva, Switzerland). Weight-for-length/height (WHZ), height-for-age (HAZ), weight-for-age (WAZ), and BODY MASS INDEX-for-age (BAZ) Z-scores were calculated for children with complete anthropometric data. Supine length was used for children <24 months and standing height for ≥24 months. Data with biologically implausible Z-scores, as defined by WHO filters, and missing values were excluded from analysis. Comparisons between cohorts used Welch’s *t*-test for mean Z-scores and the chi-square test for the proportion of children with Z-scores below −2 SD (moderate/severe malnutrition).

### 2.6. Sample Collection

For the first cohort, a previously published study’s database was used [5]. For the second cohort, patients presented to emergency departments or were hospitalized at participating hospitals and those who met the case definition and inclusion criteria were invited to participate. A questionnaire was answered by parents or guardians, and nasal swab samples were collected.

Samples were placed in viral transport media, frozen at −70 °C, and then sent to the Infectious Diseases Research Laboratory, Faculty of Medicine, UNAM, where they were stored at −70 °C until further processing.

### 2.7. Respiratory Virus Detection via Multiplex PCR

For the first cohort, samples were processed using the Anyplex RV16 multiplex RT-PCR assay (Seegene, Seoul, South Korea). For the second cohort, samples were processed using the Allplex Respiratory Full Panel multiplex RT-PCR assay (Seegene, Seoul, South Korea). Viral detection followed the manufacturer’s protocols. RNA extraction was automatically performed using Seegene’s STARMag 96 × 4 Universal Cartridge kit. A volume of 50–60 μL of sample was added to 96-well plates, which were then processed in the Bio-Rad C1000 Thermal Cycler for real-time multiplex PCR. These assays amplify and detect nucleic acids from various pathogens/subtypes including influenza A (H1, H1N1, H3), influenza B, parainfluenza (1, 2, 3, 4), adenovirus, bocavirus, respiratory syncytial virus (A, B), metapneumovirus A/B, coronavirus (NL63, 229E, OC43), rhinovirus, enterovirus for the first cohort. For the second cohort, additional detection included *Mycoplasma pneumoniae*, *Bordetella pertussis*, *Bordetella parapertussis*, *Chlamydia pneumoniae*, *Haemophilus influenzae*, *Streptococcus pneumoniae* and *Legionella pneumophila*. The software automatically interpreted amplification signals in real time, producing reports for the 26 pathogens and the internal control (IC).

### 2.8. Statistical Analysis

Normality of continuous variables was assessed using the Shapiro–Wilk test. Variables with a normal distribution were summarized as mean ± standard deviation (SD) and compared using Student’s *t*-test. IBM^®^ SPSS Statistics version 24 (IBM Corp., Armonk, NY, USA) was used. Categorical variables between the two cohorts were compared using the χ^2^ test or Fisher’s exact test, as appropriate. A *p* value < 0.05 was considered statistically significant. Respiratory rate was analyzed as a continuous variable without age adjustment, given the descriptive objective of the study. To compare the number of coinfections between cohorts, bacterial results from the second cohort were excluded from analysis because the first cohort only included viral results.

Additionally, logistic regression was applied to evaluate whether risk factors associated with pneumonia, as reported in the literature, were correlated with the development of severe pneumonia, as defined by the WHO classification [9]. The incidence density of RSV was defined as the number of new RSV-positive pneumonia cases per 10 new pneumonia admissions per month, standardized by the number of children at risk during each observation period.

## 3. Results

### 3.1. Demographic and Clinical Characteristics

The databases of children with RSV infections between March 2010 and August 2013, and July 2021 and March 2023, were compared. The population characteristics are shown in Table 1.

In the first cohort, from March 2010 to August 2013, a total of 1404 children under 5 years old with a clinical and/or radiological diagnosis of community-acquired pneumonia were recruited, of whom 531 (37.82%) tested positive for RSV and had complete demographic data.

In the second cohort, from August 2021 to March 2023, 579 children under 5 years old with a clinical and/or radiological diagnosis of community-acquired pneumonia were recruited, of whom 248 (42.83%) tested positive for RSV.

The mean age (±standard deviation (SD)) of participants was 9.97 ± 10.91 vs. 15.14 ± 15.97 months (*p* < 0.001), 63.80% of patients (n = 339) were male in the first cohort and 50.40% (n = 125) in the second cohort, while female accounted for 36.20% (n = 192) and 49.60% (n = 123) (*p* < 0.001), in the first and the second cohort, respectively. The mean height (± SD) was 0.68 ± 0.13 m vs. 0.73 ± 0.19 m (*p* < 0.001), and the mean weight was 7.59 ± 3.31 kg vs. 8.57 ± 4.08 kg (*p* < 0.001). A total of 520 (97.9%) vs. 242 (97.60%) of the children were hospitalized. A total of 9 children (1.7%) in the 2010–2013 cohort and 2 (0.8%) in the 2021–2023 cohort required admission to the Pediatric Intensive Care Unit (PICU) (Table 1).

Among children with complete data, mean Z-scores in the 2010–2013 cohort were −0.27 (SD 2.43) for WHZ, −2.37 (SD 2.12) for HAZ, −3.05 (SD 2.35) for WAZ, and −0.48 (SD 2.59) for BAZ. In the 2021–2023 cohort, no significant differences were observed for WHZ (0.08 ± 8.79; *p* = 0.64), BAZ (0.08 ± 7.52; *p* = 0.39) or HAZ −2.42 (SD 1.95).

In infants admitted between 2010 and 2013, the mean respiratory rate (±SD) was 52.56 ± 13.37 breaths per minute, and 94.2% (n = 500) presented with a cough. Regarding signs of respiratory distress, intercostal retraction was the most frequently observed (73.60%), followed by thoracoabdominal dissociation (58.80%) and nasal flaring (48.60%). Most patients exhibited an interstitial radiographic pattern (40.70%) (Table 2). In contrast, infants of the 2021–2023 cohort had a mean respiratory rate (± SD) of 40.90 ± 24.17 breaths per minute, with 88.70% (n = 220) presenting with a cough. Intercostal retraction was the most frequently observed sign of respiratory distress (60.10%), followed by thoracoabdominal dissociation (35.50%) and xiphoid retraction (30.60%). Most patients exhibited an interstitial radiographic pattern (55.60%). Significant differences were observed in most clinical characteristics when compared to the 2010–2013 period Table 2.

Antibacterial use during hospitalization was documented in 237/248 (95.6%) records for the 2021–2023 cohort; among these, 112/237 (47.3%) patients received at least one antibiotic course.

An important finding was the shift in the age of presentation from 10 to 15 months. Additionally, there were more RSV B cases during the 2021–2023 period compared to the 2010–2013 period (58.5% vs. 3.8%, respectively). There were more co-infections (patients with RSV and one or more different viruses) during the 2021–2023 period (60% vs. 39%, *p* < 0.001). Among the 60% RSV-positive cases with viral co-infection in the 2021–2023 cohort, human rhinovirus (HRV) was the most frequent accompanying virus, detected in 133 cases (89.3%), followed by human bocavirus in 17 (11.4%), human metapneumovirus in 6 (4.0%), adenovirus in 5 (3.4%), enterovirus (HEV) in 4 (2.7%), and influenza A (H3) in 2 (1.3%) cases. Percentages exceed 100% because some patients had more than one additional viral pathogen detected. According to the WHO pneumonia classification [9], 60.10% of cases were classified as severe during this period, compared to 74.8% in the 2010–2013 period. A statistically significant decrease in pneumonia severity was observed between the two cohorts (*p* < 0.001). Table 1 and Table 2.

### 3.2. Risk Factors in Infants with Severe RSV Pneumonia

Regarding the risk factors previously associated with severe pneumonia, higher proportions were observed in the first cohort for absence of breastfeeding (41% vs. 24.8%), biomass exposure (17% vs. 8%), comorbidities (32% vs. 21%), lack of influenza vaccination (75.8% vs. 72.5%), incomplete vaccination schedule (45.3% vs. 47%), and immunocompromise (5% vs. 3.3%). Conversely, household tobacco smoke exposure (36% vs. 48%) and daycare attendance (5.5% vs. 10%) were more frequent in the second cohort.

Despite these variations, the odds ratios and 95% confidence intervals overlapped for all factors, indicating no significant differences in the risk of severe pneumonia between cohorts (Table 3).

### 3.3. Seasonality

**Figure 1:**
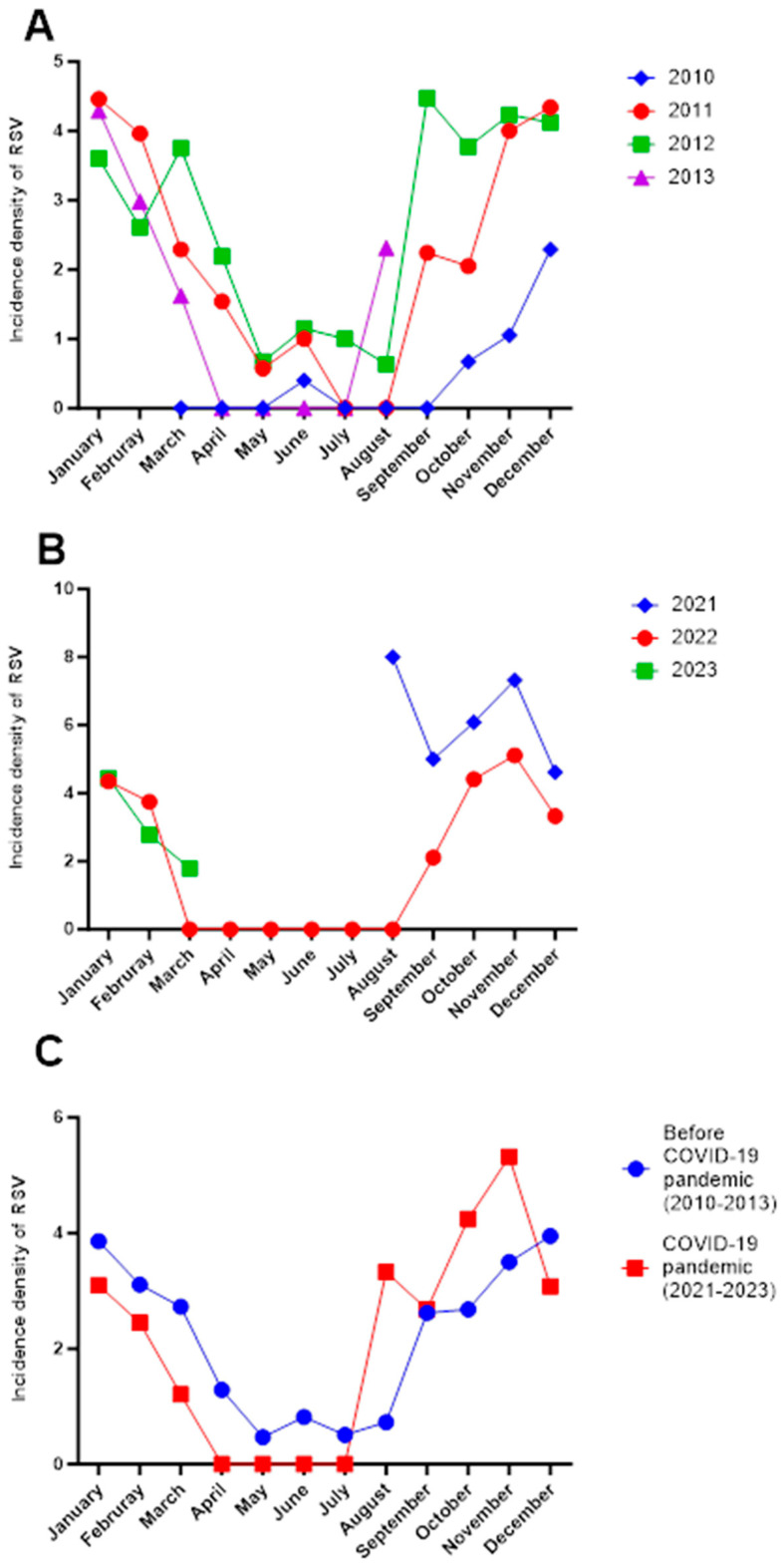
Incidence density of pneumonia in Mexican children before and during the SARS-CoV2 pandemic. (**A**) Monthly distribution of the incidence density of RSV in the period 2010–2013 by year in Mexican children with pneumonia (before the COVID-19 pandemic). (**B**) Monthly distribution of the incidence density of RSV in the period 2021–2023 by year in Mexican children with pneumonia (during the second half of the COVID-19 pandemic). (**C**) Comparison of the mean monthly incidence density of RSV cases between 2010 and 2013 and 2021–2023 in Mexican children with pneumonia. The incidence density was calculated as cases of RSV per 10 new cases of pneumonia for each month in every year. No data were available between January and July 2021 or after April 2023, as patient recruitment for the 2021–2023 cohort began in July 2021 and concluded in April 2023, corresponding to the active period of data collection. Additionally, the pandemic period in Mexico extended from March 2020 to April 2023. From the onset of the pandemic in early 2020 until July 2021, the number of pneumonia cases decreased; it was not until July 2021 that respiratory viruses began to circulate again.

With respect to the seasonal pattern of RSV, in the first cohort (2010–2013), the highest incidence was observed between September and March, with the peak in December, and low activity from April to August in all years (Figure 1A).

On the other hand, in the second cohort (2021–2023), during the second year of the COVID-19 pandemic, there was a peak incidence observed in August and October 2021, with the highest incidence in August (8 vs. 4 per 10 new cases per month) compared to 2022 or the 2010–2013 period. A similar phenomenon occurred in 2022, with no circulation of RSV from March to August, followed by a resurgence in September (Figure 1B).

When comparing both cohorts, a shift was observed in RSV circulation, represented by an increase in the number of cases from August onward, corresponding to the summer season in the 2021–2023 period compared to the 2010–2013 period Figure 1C.

## 4. Discussion

This study compared two cohorts of hospitalized children with RSV-associated pneumonia in Mexico, revealing significant epidemiological and clinical changes after the COVID-19 pandemic. The main findings included an increase in the mean age of affected children, a marked shift in RSV seasonality toward summer and autumn months, a predominance of RSV-B subtype, and a higher frequency of viral and bacterial co-infections during the 2021–2023 period. Conversely, the proportion of severe pneumonia cases significantly decreased compared with the 2010–2013 cohort. Together, these results suggest that the pandemic and its related public health measures had a lasting impact on RSV transmission patterns and clinical presentation in pediatric populations.

Before the COVID-19 pandemic, most children under 2 years old experienced RSV-related infections, particularly during their first year of life, which helped them develop some degree of immunity against severe disease in future encounters with the virus. However, in this study, we observed an increase in the average age of RSV-positive cases in the period from 2021 to 2023 compared to previous reports (10 vs. 15 months), reflecting a trend of the virus to infect older children. Several authors have hypothesized that the marked reduction in RSV circulation during the COVID-19 pandemic may have led to increased susceptibility among older infants and toddlers once community transmission resumed (“immunity debt”). However, real-world data confirming a higher clinical severity—such as increased need for respiratory support, PICU admission, or mortality—remain limited [1].

The lower respiratory rate observed in the 2021–2023 cohort is likely attributable to the higher mean age of patients, as normal respiratory rates decrease with age. Age-adjusted analyses will be considered in future multicenter studies.

Regarding the RSV subtypes, the literature suggests that RSV-A is the most common, accounting for 60% of reported infections. However, recent years have seen an increase in RSV B infections [10]. In our study, RSV-B infections rose during the pandemic period compared to the 2010–2013 period (58.5% vs. 3.8%). This shift was also reported in Australia, where RSV-B predominated in 2021 following the reintroduction of RSV circulation [11]. Although some studies have suggested potential differences in clinical severity between RSV subtypes, current evidence remains inconclusive and largely suggestive rather than definitive. Our data showed a decrease in the severity of reported cases during the 2021–2023 period (60.1%) compared to previous years (74.8%), as indicated by a lower percentage of patients showing signs of respiratory distress and the number requiring ICU admission. This could reflect the older average age of infants admitted with RSV infection during this period or possibly the presence of a higher percentage of risk factors in the 2010–2013 cohort, such as lack of breastfeeding, biomass use, comorbidities, absence of influenza vaccination, incomplete vaccination schedules and immunocompromised status.

Additionally, exposure to household smoke has been described as a factor that increases the incidence of pneumonia in children and susceptibility to acute respiratory infections (ARIs). A study showed a 58% increase in the risk of childhood ARIs in households with in-house smoking compared to non-smoker households (OR 1.58; 95% CI 1.02 to 2.47, *p* = 0.04). Cigarette smoke contains various harmful particles, including polycyclic hydrocarbons, carbon monoxide, nicotine, nitrogen oxides, and acrolein, which can damage ciliated epithelial cells, reduce mucociliary clearance, and suppress phagocyte activity and bactericidal effects, thereby compromising the pulmonary defense system [12,13]. However, our data did not show significant results indicating that smoking was a risk factor for severe pneumonia.

Furthermore, having an incomplete vaccination schedule could be associated with a more severe disease course. Currently, RSV vaccines are not available in Mexico, nor are they part of the Mexican immunization program. Therefore, while a complete vaccination schedule may not directly impact RSV infections, it could affect the frequency of co-infections, thereby influencing disease severity. Some studies have suggested that specific viral interactions and virus co-infections can alter the severity and prognosis of respiratory infections, either for better or worse [14]. For instance, Zhang et al. reported that adult patients with influenza and RSV co-infections had worse outcomes than those infected only with influenza or RSV [15].

In this study, we explored the absence of the influenza vaccine and its potential association with viral co-infections. Notably, we observed a significant increase in the number of co-infections, with 149 cases (60%) recorded between 2021 and 2023, compared to 207 cases (39%) from 2010 to 2013. However, our findings did not align with the observation that RSV-related severe pneumonia was more prevalent before the COVID-19 pandemic. This apparent discrepancy may be explained by the older mean age of patients in the 2021–2023 cohort, as disease severity tends to decrease with age. Additionally, risk factors associated with more severe disease—such as lack of breastfeeding, biomass exposure, and comorbidities—were more frequent in the 2010–2013 cohort.

It is important to note that our analysis did not include bacterial co-infections, which have been reported to significantly increase the severity of outcomes in children with respiratory infections, leading to higher rates and longer durations of invasive ventilation (3.0% vs. 0.0%) and ICU admission (11.9% vs. 6.4%) compared to RSV-only cases [16]. Further investigation is needed to understand the implications of viral and bacterial co-infections on the severity of respiratory illnesses.

An interesting observation was the shift in RSV seasonality in Mexico toward summer and autumn months, possibly linked to PHSMs implemented during the COVID-19 pandemic, such as mask use, hand hygiene, and social distancing. A study in Tokyo showed a 97.9% reduction (95% CI: 94.8–99.2%) in RSV activity during the implementation of non-pharmaceutical interventions aimed at controlling SARS-CoV-2 spread [1]. However, a delayed RSV epidemic emerged once these measures were lifted. In this study, this shift in seasonality was particularly noticeable in August 2021, when a sharp increase in RSV cases occurred, mirroring the resumption of pre-COVID-19 pandemic social behaviors driven by the immunological debt caused by prolonged social isolation. This trend is consistent with global reports, suggesting that extended social distancing can increase susceptibility to infections other than SARS-CoV-2, amplifying the risk of large RSV outbreaks [17].

The prevalence of low height-for-age Z-scores was high in both cohorts; this may reflect chronic nutritional deficiencies exacerbated by socioeconomic constraints during the pandemic, as well as the impact of delayed healthcare access. The exclusion of missing anthropometric data and the application of WHO plausibility filters ensured that these results are robust and unbiased. Future studies should incorporate complete anthropometric evaluations across all sites to better elucidate the relationship between nutritional status and RSV severity.

Although specific preventive measures such as the monoclonal antibody nirsevimab and maternal RSVpreF vaccination have recently been approved in several countries, these strategies were not yet available in Mexico during the 2021–2023 study period. Their eventual implementation could have a significant impact on RSV epidemiology and hospitalization rates in the coming years.

Finally, viral co-infections were more frequently detected in the 2021–2023 cohort, likely reflecting the relaxation of pandemic-related nonpharmaceutical interventions and differences in diagnostic panels used (the Allplex™ system detects a broader range of respiratory pathogens than Anyplex™). These factors should be considered when comparing frequencies across periods. Given the limited emphasis on co-infections in the Results and their multifactorial nature, this finding was interpreted cautiously.

Regarding the limitations of our study, only infants whose parents or guardians provided consent were included, so not all patients meeting the pneumonia diagnostic criteria were analyzed. This was a convenience sample rather than a randomized one. Additionally, we analyzed two cohorts from different time periods, and not all participating hospitals were included in both periods. Information on invasive mechanical ventilation was not available in either dataset. Moreover, risk factor information, such as household smoking, was based on parental reports and could be subject to recall bias. Finally, the observational design limits causal inferences, and unmeasured confounders may have influenced the observed associations.

## 5. Conclusions

RSV remains a significant viral pathogen in the pediatric population associated with pneumonia. Our study highlighted notable changes in the characteristics of children admitted with RSV during the pandemic compared to previous years, including an increase in the mean age of affected children from 10 to 15 months. Moreover, in 2021, RSV activity started earlier than usual (from August), with heightened activity in October, November, and December. This underscores the importance of continuous surveillance to establish preventive measures against this virus.

## Figures and Tables

**Table 1:** Demographic characteristics of the subjects.

Characteristics	2010–2013 (n = 531)	2021–2023 (n = 248)	*p*
Sex			
Female, n (%)	192 (36.20)	123 (49.60)	
Male, n (%)	339 (63.80)	125 (50.40)	0.001 **
Age (months, $\bar{x}$ ± SD)	9.97 ± 10.91	15.14 ± 15.97	0.001 *
Height (meters, $\bar{x}$ ± SD)	0.68 ± 0.13	0.73 ± 0.19	0.001 *
Weight (kilograms, $\bar{x}$ ± SD)	7.59 ±3.31	8.57 ± 4.08	0.001 *
Z score weight for height	−0.27 ± 2.43	0.08 ± 8.79	0.64
Z score height for age	−2.37± 2.12	−2.42 + 1.95	0.83
Z score weight for age	−3.05 ± 2.35	−3.64 ± 3.46	0.24
Z score BMI for age	−0.48 ± 2.59	0.08 ± 7.52	0.39
Hospital Area			
Ambulatory, n (%)	2 (0.4)	4 (1.60)	0.116 **
Hospitalization n (%)	520 (97.90)	242 (97.60)	
Intensive care unit n (%)	9 (1.70)	2 (0.80)	

* Student’s *t*-test and ** Pearson’s Chi-square were used to assess statistical differences between the characteristics of both cohorts. SD: Standard Deviation. *p* value < 0.05 is statistically significant.

**Table 2:** Clinical Characteristics of infants hospitalized with RSV infection.

Characteristics	2010–2013 (n = 531)	2021–2023 (n = 248)	*p*
Temperature (°C, $\bar{x}$ ± SD)	37.30 ± 1.91	36.83 ± 0.75	0.001 *
Respiratory rate (bpm, $\bar{x}$ ± SD)	52.56 ± 13.37	40.90 ± 24.17	0.001 *
Cough, n (%)	500 (94.20)	220 (88.70)	0.005 **
Thoracoabdominal dissociation, n (%)	312 (58.80)	88 (35.50)	0.001 **
Intercostal retraction, n (%)	391 (73.60)	149 (60.10)	0.001 **
Xiphoid retraction, n (%)	181 (34.10)	76 (30.60)	0.341 **
Nasal flaring, n (%)	258 (48.60)	70 (28.20)	0.001 **
Subtype			
RSV A, n (%)	511 (96.2)	103 (41.5)	0.001 **
RSV B, n (%)	20 (3.8)	145 (58.5)	
Co-infections, n (%)	207 (38.98)	149 (60.08)	0.001 **
WHO Severity score			
Severe, n (%)	397 (74.80)	149 (60.10)	0.001 **
Non-severe, n (%)	134 (25.20)	99 (39.90)	
Radiographic pattern			
Interstitial, n (%)	216 (40.70)	138 (55.60)	
Mixed, n (%)	45 (8.50)	25 (10.10)	
Lobar, n (%)	53 (10.0)	18 (7.30)	
Micro and/or macronodular, n (%)	61 (11.50)	16 (6.50)	0.001 **
Multiple foci, n (%)	92 (17.30)	6 (2.40)	

Student’s *t*-test * and Pearson’s Chi-Square ** tests were used to assess statistical differences between the characteristics of both cohorts. SD = Standard Deviation. *p* value < 0.05 is statistically significant.

**Table 3:** Risk factors in children with severe RSV pneumonia during the two study periods.

Risk Factors	2010–2013	(n = 531)		2021–2023	(n = 248)	
	S (n = 397) n (%)	NS (n = 134) n (%)	OR (95% CI)	S (n = 149) n (%)	NS (n = 99) n (%)	OR (95% CI)
Absence of breastfeeding	165 (41)	60 (44)	1.140 (0.768–1.692)	37 (24.8)	23 (23.2)	0.916 (0.505–1.663)
Domestic smoking	145 (36)	47 (35)	1.065 (0.707–1.604)	72 (48.3)	47 (18.9)	1.035 (0.622–1.721)
Biomass use	70 (17)	34 (25)	0.630 (0.395–1.004)	12 (8)	12 (12.1)	0.635 (0.273–1.477)
Daycare attendance	22 (5.5)	7 (5.2)	1.064 (0.444–2.551)	15 (10)	6 (6)	1.735 (0.649–4.637)
Presence of comorbidities	129 (32)	32 (23.9)	1.534 (0.979–2.404)	32 (21)	13 (13.1)	1.809 (0.897–3.651)
Absence of influenza vaccine	301 (75.8)	92 (68.5)	0.699 (0.454–1.075)	108 (72.5)	75 (75.7)	1.186 (0.662–2.126)
Incomplete vaccination schedule	180 (45.3)	52 (38.8)	0.764 (0.513–1.140)	70 (47)	37 (37.3)	0.674 (0.401–1.132)
Immunocompromised	20 (5)	8 (5.9)	0.836 (0.359–1.944)	5 (3.3)	2 (2)	1.684 (0.320–8.856)

The Odds Ratio with 95% Confidence Interval was calculated for the probability of occurrence of severe pneumonia for each risk factor. S = Severe pneumonia, NS = Non-severe pneumonia, OR = Odds Ratio, 95% CI = Confidence interval.

## Data Availability

De-identified individual-level data for both cohorts and the accompanying data dictionary are available from the corresponding author upon reasonable request and subject to institutional and ethics approval due to patient privacy considerations. The 2010–2013 cohort was curated from the database of the previously published multicenter study by Wong-Chew et al. [5], for which informed consent included authorization for future data use for up to 20 years.
